# Feasibility and acceptability of the Trans-theoretical Model-based advance care planning for community-dwelling older adults with chronic diseases in China: a randomized controlled trial

**DOI:** 10.3389/fpubh.2025.1686912

**Published:** 2025-11-26

**Authors:** Siyuan Feng, Jufeng Chen, Mingli Zhao, Yijia Zhuo, Xinyue Zhao, Xue Wang, Beibei Qiao

**Affiliations:** 1 Shanghai Fifth People's Hospital, Affiliated to Fudan University, Shanghai, China; 2 Center of Community-Based Health Research, Fudan University, Shanghai, China; 3 Shanghai Chest Hospital, Affiliated to Shanghai Jiaotong University School of Medicine, Shanghai, China; 4 School of Nursing and Health, Zhengzhou University, Zhengzhou, China

**Keywords:** the Trans-theoretical Model, advance care planning, older adults, chronic disease, aging

## Abstract

**Background:**

Advance care planning (ACP) has emerged as a structured process to honor participants’ autonomy in medical decision-making. Evidence indicates that participation in ACP among community-dwelling older adults with chronic conditions is associated with enhanced medical autonomy, improved quality of life, and reduced decisional burden for family caregivers. However, given cultural nuances specific to China, ACP implementation remains limited and underinvestigated, underscoring the necessity of feasibility studies to guide subsequent research and practice.

**Design:**

A 6-week feasibility randomized controlled trial was conducted in community settings, with 44 older adults enrolled in Zhengzhou, China. Methods: A randomized controlled design was employed. A total of 44 community-dwelling older adults with chronic diseases meeting the inclusion criteria were recruited and randomly assigned to either the intervention group (*n* = 22) or the control group (*n* = 22). The intervention, delivered between August and September 2024, consisted of a Trans-theoretical Model (TTM)-guided program designed to enhance ACP participation. Data were collected at four time points: baseline (T0), immediately post-intervention (T1), 1-month follow-up (T2), and 3-month follow-up (T3) with validated questionnaires used to assess ACP knowledge, attitudes, and participation. Statistical analyses were performed using SPSS 26.0, with primary outcomes including scores for ACP knowledge, attitudes, and participation to evaluate intervention efficacy.

**Results:**

Of the 44 enrolled participants, 4 were lost to follow-up, resulting in an attrition rate of 9.1% and a final sample size of 40. At baseline, all participants were in the precontemplation stage of ACP engagement. Immediately post-intervention, behavioral changes toward ACP participation were demonstrated by 17 participants (85%) in the intervention group. The intervention group exhibited significantly higher scores in ACP knowledge, attitudes, and participation at T1, T2, and T3 (all *p* < 0.001). For participation scores, significant effects were observed for group (Wald *χ*^2^ = 25.965, *p* < 0.001), time (Wald *χ*^2^ = 454.226, *p* < 0.001), and group time interaction (Wald *χ*^2^ = 504.720, *p* < 0.001).

**Conclusion:**

The TTM-based ACP intervention shows promise in potentially improving ACP knowledge, attitudes, self-efficacy, and readiness among community-dwelling older adults with chronic diseases in this feasibility trial. Further research is warranted to highlight the need for larger-scale, multi-center trials with follow-up to further validate the intervention’s efficacy.

## Introduction

Amidst accelerating demographic aging and rapid technological advancement, the healthcare industry is facing both unprecedented opportunities and complex challenges (1). The issue of overtreatment among participants with end-stage chronic diseases has become increasingly pronounced (2). This not only fails to enhance participants’ quality of life but also exacerbates the physical, psychological, and economic burdens on their families (3). Studies indicate that approximately 1/3 of participants wish to discuss their future medical and nursing-related decisions with family members and healthcare providers, hoping to obtain secure their understanding (4); however, most are unable to fulfill this desire. Advance care planning (ACP) serves as an effective solution to this predicament. It is defined as a process that enables adults of any age or health status to share their personal values, future medical preferences, and care goals (5). ACP comprises three core elements: (1) Surrogate Decision Makers (SDM); (2) discussions regarding personal values and preferences for future medical options; (3) Advance Directives (ADs) (6). Research has confirmed that ACP not only protects participants’ medical autonomy but also helps individuals receive care aligned with their treatment and nursing -related preferences, thereby improving their future quality of life (7). Currently, ACP is primarily implemented among end-stage participants in hospital settings, with demonstrated effectiveness. Nevertheless, clinical practice reveals that the old end-stage participants have lost their decision-making capacity, rendering ACP discussions untimely (8). Consequently, the intervention target population for intervention has shifted from hospitals to communities.

In 2014, the American Medical Association (AMA) proposed that older adult community residents are an important target group for promoting the development of ACP from the perspectives of sustainability, cost-effectiveness, and social diffusion (9). Currently, relevant studies have confirmed the effectiveness of ACP interventions in older adult participants with chronic diseases (10, 11). Discussions with older adults focused on end-of-life medical care have been shown to alter their attitudes toward ACP and increase the signing rate of advance directives (12, 13). As research advances, an increasing number of scholars recognize that ACP constitutes a health behavior, and the Trans-theoretical Model (TTM) can reflect stages of individual behavior, enabling more scientific evaluation of ACP participation and providing a theoretical basis for stage-based changes in ACP behavior (14). Therefore, the stage-based behavioral changes described by TTM provide a novel approach to evaluating and enhancing ACP participation. The research team led by Fried from Yale University has continuously focused on the application of TTM-based ACP (ACP-T) intervention programs in community settings, with results demonstrating that TTM interventions facilitate changes in participants’ ACP behaviors (15). Sudore et al. conducted several randomized controlled trials investigating the impact of the “PREPARE website”—developed based on TTM—on the signing rate of ACP participation among older adults (16). The patient-facing PREPARE program increased documentation of advance care planning and patient-reported engagement in ACP (17). ACP-T programs are increasingly utilized among older adult participants to promote ACP implementation.

## Background

In China, shaped by traditional cultural norms and filial piety values, family members of participants commonly adopt a medical orientation centered on maximizing the patient’s lifespan (18). Studies have shown that Chinese participants and their families have a low consistency in AD (19). Intensive life-sustaining interventions are frequently opted for, which, paradoxically, lead to compromised quality of life for the participants (20, 21). As the hierarchical healthcare system evolves, community medical institutions have been providing appropriate, comprehensive, and continuous nursing interventions and support to participants, facilitating a gradual transition of treatment settings from hospitals to communities for individuals with chronic conditions (22). Communities have emerged not only as pivotal healthcare service hubs for older adults with chronic diseases but also as increasingly critical venues for ACP implementation (23). In 2021, Thailand’s Office of the National Health Commission launched ACP and formed two key bodies: one tasked with creating a national ACP form and standard operating procedures, and another steering committee to oversee ACP’s nationwide rollout in community (24)_._ Among respondents in Singapore, those who participated in ACP-related activities (e.g., making a will, establishing a lasting power of attorney, sharing end-of-life care preferences with physicians, or communicating such wishes to family/loved ones) had significantly higher ACPES scores (25). Consequently, leveraging communities as the platform for ACP development in China confers distinct advantages. However, currently, ACP implementation in China is primarily conducted among participants with severe diseases such as cancer. To date, no systematic ACP-T intervention has been implemented among community-dwelling older adults. The ACP-T intervention lies in its targeted approach to addressing the critical gap in systematic, community-focused ACP implementation. The primary contribution of The TTM-based ACP intervention lies in its targeted approach to addressing the critical gap in systematic, community-focused ACP implementation. By tailoring interventions to individuals’ dynamic readiness stages for behavior change, it effectively overcomes the limitations of one-size-fits-all strategies prevalent in traditional ACP efforts, thereby strengthening the consistency of care preferences between older adults and their families. This not only helps reduce the overuse of clinically unnecessary life-sustaining treatments that undermine quality of life but also facilitates person-centered end-of-life care aligned with patients’ authentic wishes. Additionally, it enriches practical models for community-based ACP implementation.

## Objectives

The study aimed to evaluate the effects of an ACP-T intervention of community-dwelling older adults in China, with objects on stage of the ACP behavior change of the intervention group. And whether the intervention remains effective over time. Additionally, the study investigated the effect of the ACP-T on:

- The ACP knowledge of the older people
- The ACP attitude of the older people
- The ACP engagement of older people

## Methods

### Study design

**Figure 1:**
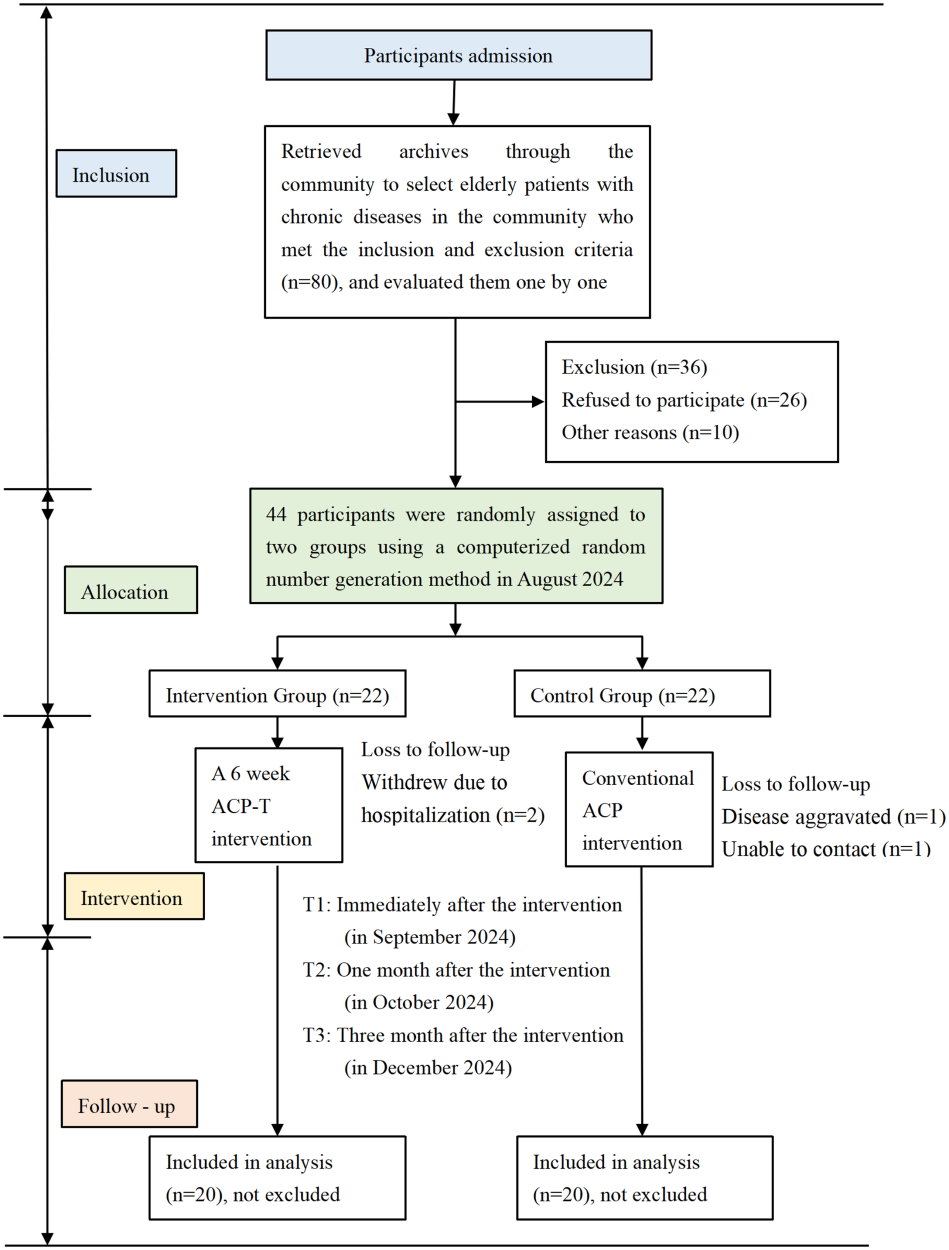
Flowchart of the participants. This is the flowchart of the ACP-T intervention.

A prospective, parallel-group, randomized controlled trial conducting a 6-week ACP intervention was conducted in Zhengzhou, China, from August 2024 to September 2024. The study flowchart is presented in Figure 1. This study was approved by the Institutional Review Board of Zhengzhou University (No. ZZUIRB2024-21). All participants provided written informed consent prior to enrollment. The intervention was aligned with the CONSORT checklist for reporting randomized trials.

### Setting and participants

We recruited community-dwelling old patients with chronic diseases from Zhengzhou. Recruitment information was disseminated via community channels. During the eligibility screening phase, older adult individuals self-assessed their eligibility (i.e., having one or more chronic diseases) and then registered to participate; their chronic disease status was subsequently verified using medical records. Two research assistants initially recruited 80 patients, and after screening against the inclusion and exclusion criteria, 44 eligible and willing participants were selected. The study flowchart is presented in Figure 1. A computer-generated random number method was employed by the research assistants who were responsible only for recruiting and grouping older adults without participating in the subsequent intervention. Using the SPSS computerized random number generator, these 44 patients were evenly allocated to two groups: one designated as the intervention group and the other as the control group. This study was conducted in communities under the jurisdiction of a community health service center in Zhengzhou, which were coordinated by the project leader.

Participants were eligible for inclusion if they met the following criteria: (1) Aged 60 years or older; (2) With at least one chronic disease; (3) With clear consciousness; (4) Without communication, reading, or writing difficulties. Exclusion criteria included: (1) Individuals with cognitive impairment relied on medical records; (2) Individuals with functional or organic mental disorders. All participants provided written informed consent prior to enrollment.

### Sample size

In the present study, the ACP participation score was used as the primary outcome measure. Sample size was estimated using the “two-sample mean comparison” method, with the formula: n = 2[(u*α* + u*β*) *σ*] ^2^ /δ^2^. For α = 0.05 (two-tailed) and β = 0.10, the corresponding values were uα = 1.96 and uβ = 1.282., the corresponding values were uα = 1.96 and uβ = 1.282. Based on preliminary survey data from our research team, the mean ACP participation score among participants was 48.20 ± 12.75, with an estimated standard deviation (*σ*) of 12.75 and a projected mean difference (*δ*) of 17 between the two groups. Considering a 20% attrition rate and practical communication barriers in the community—primarily driven by cultural and cognitive differences, such as traditional Chinese family values and low public awareness of ACP—the initial sample size was set at 22 participants per group, totaling 44 participants. Adhering to the methodological quality criteria for RCT design outlined by the Cochrane Collaboration, randomization was performed using computer-generated random numbers by the research assistants. Using SPSS 26.0 software, participants were randomly assigned to either the intervention group (*n* = 22) or the control group (*n* = 22).

### Theoretical framework

**Figure 2:**
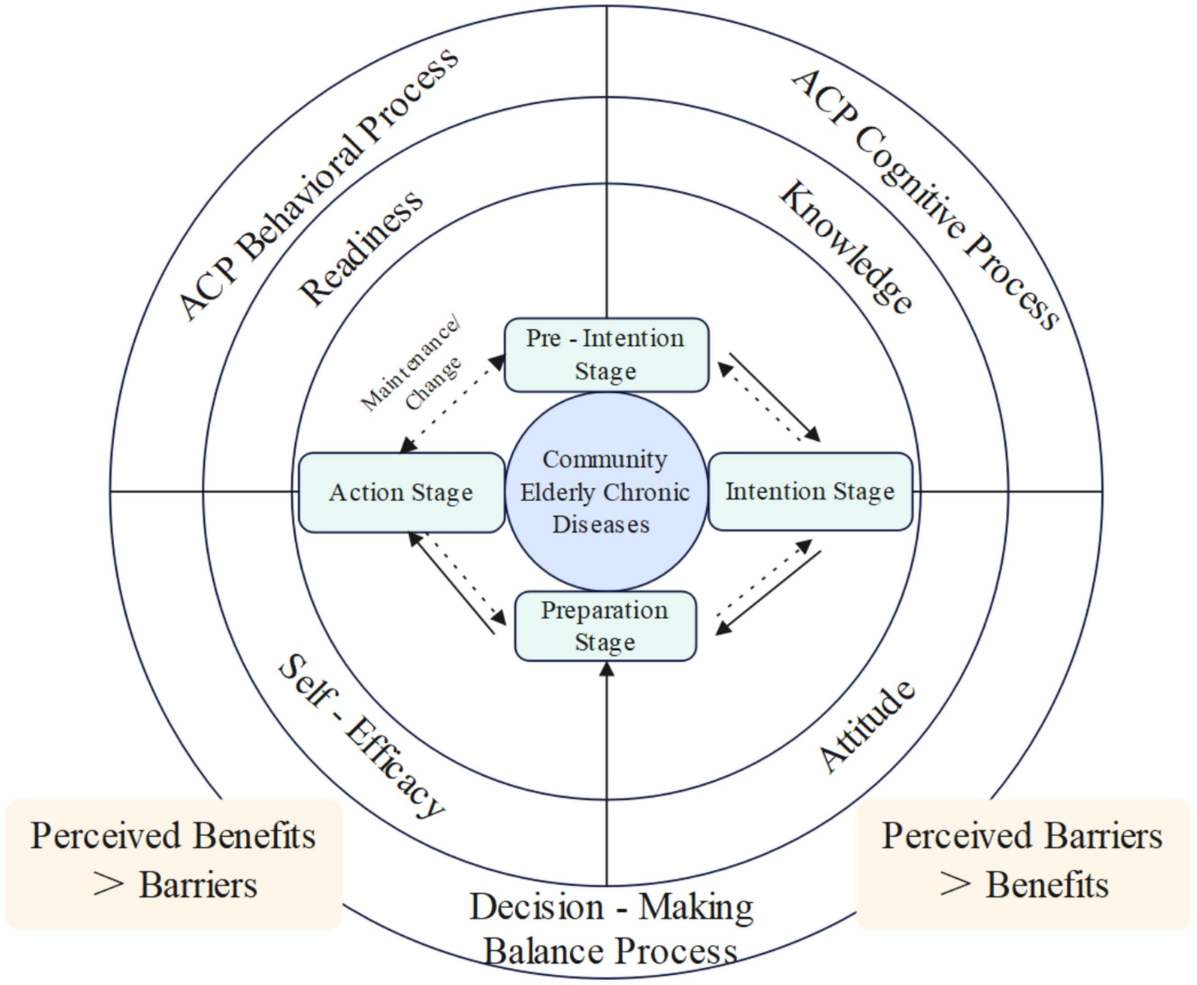
Theoretical framework. This is the trans-theoretical framework of this ACP-T intervention.

The ACP intervention program developed in this study for community-dwelling older adults with chronic diseases is based on the Trans-theoretical Model (Figure 2), which encompasses four stages: precontemplation, contemplation, preparation, action (25).

### Intervention

The research procedures were structured as follows: (1) A multidisciplinary team was established, composed of one project supervisor, three research team members, and four community healthcare providers, all tasked with implementing the intervention. (2) The community health service center was engaged to recruit older adults with chronic diseases. Eligible participants were identified, provided with written informed consent, and completed the baseline assessment (T0). (3) A standardized one-week ACP training program was delivered to the intervention team to ensure proficiency in ACP communication skills and adherence to the protocol. (4) Intervention Implementation: The six-week ACP-T intervention commenced in August 2024, following the training completion.

The Intervention Group (IG) mainly includes Group lectures and Individual Interviews. The group Lectures focus on ACP knowledge education combined with PPT presentations and video screenings; Individual Interviews cover topics such as patients’ disease experiences, life values, and medical decision-making preferences. Motivational interviewing strategies were adopted to guide patients in in-depth participation in ACP discussions. Participants in the Intervention Group received the ACP-T intervention (see Table 1). ACP brochures are provided throughout the 1st to 6th interventions; and the document My Five Wishes is distributed during the final intervention. During the pre-intention stage (Week 1), ACP knowledge manuals were distributed to deliver foundational information on ACP, aimed at enhancing participants’ cognitive understanding of the concept. In the contemplation stage (Weeks 2–3), educational videos were shown to illustrate the concept of dignified death within the Chinese cultural context, supplemented by detailed explanations of ACP medical decision-making processes and participation protocols. During the preparation stage (Weeks 4–5), individual one-on-one interviews were conducted, where participants were encouraged to share their disease experiences through discussions of illness-related symptoms and distress. This approach was designed to strengthen their self-efficacy in ACP participation. In the action stage (Week 6), social support was provided to clarify pathways for ACP engagement, with Advance Directives (ADs) documents distributed and their completion documented.

**Table 1:** The content of the ACP-T intervention.

Intervention stage	Intervention time	Intervention theme	Intervention goal	Intervention content
Cognitive process				
Stage I Pre-intention 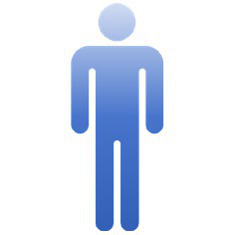	Week 1 Group Lecture	Concept Introduction Stimulating Interest	Improving ACP Knowledge Level	(1) Distribute publicity brochures to arouse patients’ interest (5 min) Explain the purpose and significance of this lecture, and maintain communication and trust; Distribute ACP knowledge brochures, briefly introduce their contents to arouse patients’ interest. (2) PPT presentation and explanation of ACP-related knowledge (20 min) Get to the core: Introduce basic ACP knowledge to enhance patients’ cognitive level, including: basic concepts of ACP, target population, and main contents (selecting medical proxies, ACP discussions, and signing advance directives). (3) Video playback (20 min) (4) Summary (5 min) Inquire about patients’ questions and provide timely answers by members of the research team;
Stage II Intention 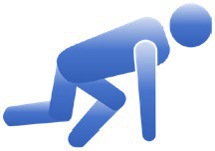	Week 2 Group Lecture	Cultural Adaptation Emotional Triggering	Contents related to traditional Chinese culture Promote emotional resonance	(1) Brief review to enhance patients’ cognition (5 min) (2) PPT presentation of traditional Chinese culture related to ACP (10 min) It mainly includes: ① understanding patients’ cultural perception; ② reducing cultural sensitivity; ③ enhancing cultural identity; ④ improving cultural adaptation: explaining the development of ACP in China, enabling patients to understand medical ethics norms and the development of ACP, and actively participating in ACP on the basis of meeting personal cultural preferences. (3) Video playing to arouse patients’ thinking (20 min) ① Why dignified death is needed (10 min); ② Enlightenment from dignified death (10 min). (4) Summary (5 min) Distribute the behavior change questionnaire to assess patients’ behavioral stages; those willing to continue participating will proceed to the next stage of intervention.
	Week 3 Group Lecture	Simplifying Procedures and Examining Life	Improve perceived benefits of ACP Enhance patients’ perceived benefits of ACP Improve awareness of medical autonomy	(1) Simplify procedures to enhance patients’ willingness to participate in ACP (10 min) This lecture discusses and communicates patients’ perceived benefits and barriers of ACP, streamlines the ACP participation process, and strengthens patients’ willingness to engage. (2) PPT presentation of ACP participation process (10 min) ① Discuss the benefits and obstacles of ACP participation; ② Share the benefits of ACP with patients; ③ Explain the ACP participation process. (3) PPT explanation of rights related to advance care planning and medical decision-making (10 min) Enhance patients’ awareness of medical autonomy and present end-of-life medical treatment options, mainly including 4 types: a. Life-support treatment: basic nursing procedures such as blood sampling, transfusion, oxygen therapy, and antibiotics; b. Life-sustaining treatment: tracheal intubation, ventilator-assisted breathing, CVC, PICC, etc.; c. Hospice care-related content: understanding symptom management to improve comfort; d. Treatment withdrawal documents: refusal of CPR and other intensive rescue measures. (4) Review (5 min) Distribute the behavior change questionnaire to assess patients’ behavioral stages; those willing to continue participating will proceed to the next stage of intervention.
Stage III Preparation 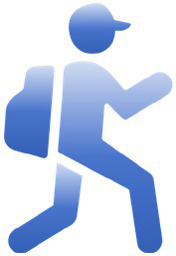	Week 4 Personal Interview	Discussing Illness and Pain Experience Exchange	Conduct in-depth communication on patients’ disease experiences and feelings Improve patients’ self-efficacy in participating in ACP	(1) One-on-one interviews to enhance understanding and communication with patients (5 min) As this topic involves the medical experiences of the patients themselves or their family members, attention should also be paid to observing changes in the patients’ emotions. (2) In-depth communication to improve patients’ ability to communicate actively and their self-efficacy (25 min) Transactional communication: Inquire about patients’ medical experiences during their illness or when accompanying relatives and friends during hospitalization, etc.; Sharing communication: Share cases of medical autonomy; share with patients how to participate in medical decision-making communication; Emotional communication: Emotional needs for future medical treatment (such as the type of company they hope to receive from family members and the care they expect from medical staff). Improve communication skills and self-efficacy through: ① question interaction; ② explanation and assistance; ③ support and encouragement; ④ sharing and negotiation; (3) End the interview (5 min) Understand the patients’ experiences and feelings about this interview, which will be recorded in a timely manner by members of the research team.
	Week 5 Personal Interview	Balanced Decision-Making and Plan Formulation	Enhance chronic disease patients’ ability to discuss ACP	(1) Brief review to strengthen patients’ cognition (5 min) ① Opening remarks: Briefly introduce oneself, explain the purpose and significance of this session, and maintain a good nurse–patient communication relationship; ② Introduce the topic: Explain the content of the fifth lecture of “Advance Directive” on the “Choice and Dignity” website to help patients develop participation plans. (2) Chronic disease patients participate in formulating advance care plans (30 min) Provide standardized participation information. ACP discussion contents: a. Understand patients’ knowledge and experience regarding their disease and health; b. Provide disease-related information, knowledge, and information on the treatment measures; c. Understand patients’ goals and preferences for future medical treatment and care; d. Understand patients’ tendencies in selecting medical agents; e. Discuss patients’ end-of-life healthcare options and potential problems; f. Provide advance directive documents and encourage patients to proactively share them with caregivers and medical staff. (3) End the interview (5 min) Distribute the behavior change questionnaire to assess patients’ behavioral stages. Those willing to continue will proceed to the next stage of intervention.
Action process				
Stage IV Action 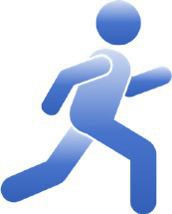	Week 6 Personal Interview	Social Support and Respecting Wills	Enhance patients’ ability to participate in ACP Provide support for patients’ advance care plans	(1) Brief review to strengthen patients’ cognition (5 min) ① Opening remarks: Briefly introduce oneself, explain the purpose and significance of participating in the advance care plan, and maintain a good nurse–patient relationship; ② Introduce the topic: Explain the content of the sixth lecture of “Advance Directive” on the “Choice and Dignity” website. (2) Chronic disease patients participate in the advance care plan (20 min) Provide professional information support and expert consultation on ACP manuals; offer psychological support and express understanding; provide social support channels, as well as relevant policies and publicity content from hospitals and communities. (3) Distribute small gifts to reinforce patients’ perceived benefits (5 min) Distribute the behavior change questionnaire to assess patients’ behavioral stages.

This is the specific content of the ACP-T intervention.

The Control Group (CG) received a 6-week conventional ACP educational intervention focused on the theme of “Choice and Dignity,” administered via group lectures. These lectures combined PPT presentations and screenings of videos about “My Five Wishes,” totaling 6 sessions (one session per week) with each session lasting 40 min.

Concurrently, throughout the implementation of the ACP-T intervention, researchers and nursing staff remained on-site to conduct observations, with the goals of promptly identifying potential risks and safeguarding participant safety. No adverse events or unintended effects were reported in either the intervention or control group throughout the study period.

### Outcome measurements

#### Advance care planning questionnaire, ACPQ

This scale was used to assess participants’ ACP knowledge and attitudes. The questionnaire consists of 4 sections with a total of 58 items (26): (1) Demographic data: 7 items; (2) Health information questionnaire: 2 items; (3) ACP knowledge questionnaire: 6 items covering content such as ACP, surrogate decision-makers, end-of-life decision-making, advance directives, power of attorney, and access channels; (4) ACP attitude questionnaire: 43 items. Among them, 27 items use a 2-option format (“Yes”/"No”) to evaluate willingness; the ACP attitude score comprises 16 items utilizing a 5-point Likert scale, with scores ranging from 1 (“strongly disagree”) to 5 (“strongly agree”). The total score ranges from 16 to 80, with higher scores indicating more positive attitudes toward ACP. The Cronbach’s *α* coefficient for this questionnaire is 0.915.

#### Advance care planning engagement survey, ACPES

The Advance Care Planning Engagement Survey (ACPES) was used to assess ACP participation and stages of behavioral change among participants. The questionnaire focuses on four ACP themes (27): (1) Designating a medical surrogate decision-maker; (2) Life values; (3) Discussing ACP decisions with family members; (4) Discussing ACP with doctors. It measures participation throughout the entire ACP behavioral process across four dimensions: cognition, contemplation, self-efficacy, and readiness, using a 5-point Likert scale with scores ranging from 1 to 5. Higher scores indicate higher levels of participants’ ACP cognition, contemplation, self-efficacy, and readiness. The overall Cronbach’s *α* coefficient for the questionnaire is 0.817, the test–retest reliability is 0.846, and the Cronbach’s α coefficients for each dimension range from 0.606 to 0.881.

### Data collection

The ACPQ and ACPES questionnaires were distributed to participants, with data collected at four specified time points: prior to intervention initiation (T0), immediately after the final intervention session (T1), one-month post-intervention (T2), and 3 months post-intervention (T3).

### Data analysis

Questionnaire data were analyzed descriptively in SPSS software (Version 26). (1) Sociodemographic characteristics of the control and intervention groups were compared using descriptive statistics. (2) Descriptive statistics were used to analyze pre- and post-intervention changes in the intervention group’s participation behaviors. (3) Independent-samples t-tests, chi-square tests, Fisher’s exact tests, and Mann–Whitney U tests assessed group equivalence in ACP knowledge, attitudes, and participation. (4) Repeated measures ANOVA or generalized estimating equations (as appropriate) compared between-group differences in intervention effects on cognition, attitudes, and participation. This statement adheres to CONSORT principles for intervention research, ensuring readers can fully evaluate the rigor and reproducibility of the study’s analytical process.

## Results

### Baseline characteristics

A total of 44 community-dwelling older adults with chronic diseases were enrolled in this study. During the intervention period, 4 participants were lost to follow-up (2 in each group): in the Control Group (CG), 1 participant withdrew due to being unreachable (attributed to personal reasons) and 1 due to deterioration in their disease condition; in the Intervention Group, both lost participants dropped out due to hospitalization for treatment. This resulted in a final sample size of 20 participants per group (CG and Intervention Group) for the final analysis. The mean age of the intervention group was (66.95 ± 9.428) years, while that of the control group was (66.2 ± 6.040) years. Comparative analysis showed that baseline characteristics were balanced between the two groups, with no statistically significant differences (all *p* > 0.05), as detailed in Table 2. Comparisons of demographic characteristics between participants, no statistically significant differences were observed (all *p* > 0.05), suggesting minimal non-response bias.

**Table 2:** Comparison of baseline data of patients.

Project	Control group (x̅±s/Median/Percentage)	Intervention group (x̅±s/Median/Percentage)	*t*/x^2^ value	*p* value
Age	66.20 ± 6.040	66.95 ± 9.428	0.300^a^	0.766
Gender			−0.902^b^	0.527
Male	12 (60.0%)	9 (45.0%)		
Female	8 (40.0%)	11 (55.0%)		
Marital status			−0.667^d^	0.717
Married	17 (85.0%)	17 (85.0%)		
Divorced	1 (5.0%)	2 (10.0%)		
Widowed	2 (10.0%)	1 (5.0%)		
Educational level			−1.048^d^	0.592
Primary school and below	1 (5.0%)	0 (0.0%)		
Junior high school	10 (50.0%)	11 (55.0%)		
Senior high school and above	9 (50.0%)	9 (45.0%)		
Religious belief			−0.360^d^	1.000
Yes	1 (5.0%)	2 (10.0%)		
No	19 (95.0%)	18 (90.0%)		
Economic income			−0.125^b^	0.723
Less than 3,000	15 (75.0%)	14 (70.0%)		
3,000 and above	5 (25.0%)	6 (30.0%)		
Living arrangement			−0.229^d^	1.000
Living alone	2 (10.0%)	3 (15.0%)		
Spouse/Children	18 (90.0%)	17 (85.0%)		
Self-rated health status			−0.400^d^	0.819
Poor	1 (5.0%)	2 (10.0%)		
Fair	11 (55.0%)	11 (55.0%)		
Good	8 (40.0%)	7 (35.0%)		
Number of current diseases			−0.107^b^	0.744
1 Disease	7 (35.0%)	8 (40.0%)		
2 or more diseases	13 (65.0%)	12 (60.0%)		
1. ACPQ cognitive level	46.55 ± 3.203	47.60 ± 4.297	0.876^a^	0.386
ACP knowledge	6.25 ± 1.164	6.90 ± 0.912	1.966^a^	0.570
ACP attitude	40.30 ± 3.181	40.70 ± 4.305	0.334^a^	0.740
2. ACPES participation degree	36.00 (35.00, 39.00)	36.50 (34.00, 40.50)	0.014^e^	0.989
Knowledge dimension	2.00 (2.00, 2.00)	2.00 (2.00, 2.00)	0.424^e^	0.820
Reflection dimension	3.00 (3.00, 3.00)	3.00 (3.00, 3.00)	−0.471^e^	0.785
Self-efficacy dimension	14.00 (12.00, 16.75)	14.00 (13.00, 16.00)	0.151^e^	0.883
Readiness dimension	17.00 (17.00, 17.00)	17.00 (17.00, 17.00)	−0.058^e^	0.968

This table is the comparison of the baseline data of the patients. a: Independent-samples t-test; b: Pearson’s χ^2^ test; c: Continuity-corrected χ^2^ test; d: Fisher’s exact test; e: Mann–Whitney U test.

### Stage of the ACP behavior change of the intervention group

**Figure 3:**
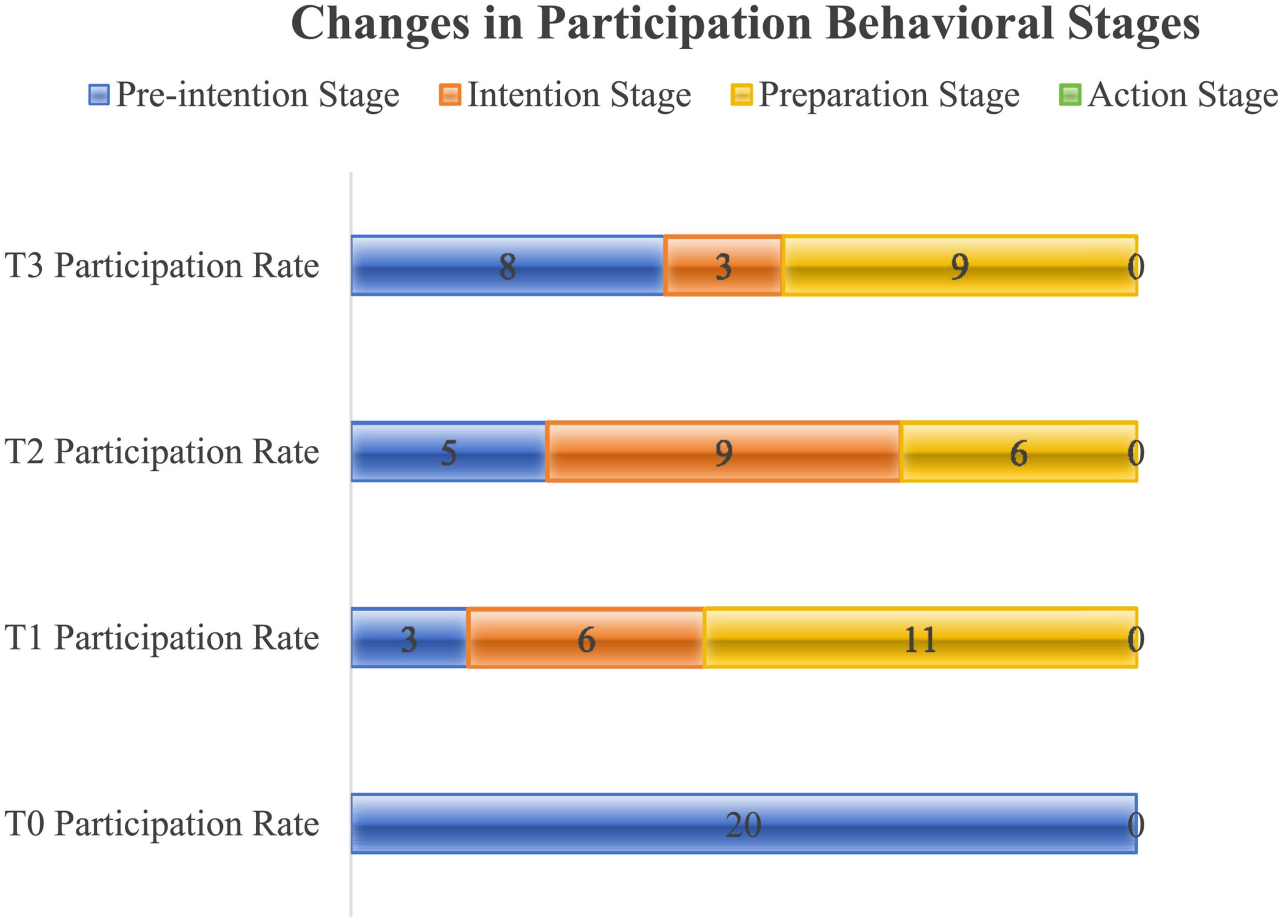
Changes in participation behavioral stages. This is the changes in participation behavioral stages of the intervention group.

Figure 3 illustrates that prior to the intervention, all 20 participants in the intervention group were in the precontemplation stage. Of these, 17 participants (85%) exhibited behavioral changes: 6 participants (30%) transitioned to the contemplation stage, while 11 participants (55%) advanced to the preparation stage in terms of ACP participation.

### Comparison of ACP knowledge scores between the two groups

Analysis of ACP knowledge scores across time points revealed no significant difference between the two groups at T0 (before intervention) (*p* > 0.05). However, statistically significant differences in ACP knowledge scores were observed at T1, T2, and T3 (*p* < 0.05), as detailed in Table 3. For inter-group and intra-group effects at each time point, there was no statistically significant difference in ACP knowledge scores between the two groups (*p* < 0.001). Additionally, a significant interaction effect between time and group was identified for ACP knowledge scores in both groups (*p* < 0.01). This means that the effect of the time factor is related to the differences in the intervention content between groups, and the effect of the intervention factor on the ACP knowledge level scores varies with the passage of time.

**Table 3:** ACP knowledge scores of patients in both groups.

Grouping	Before intervention	After Intervention			Between-group effect *F (P)*	Within-group effect *F (P)*	Interaction effect *F (P)*
		Immediate post-intervention	One month post-intervention	Three months post-intervention			
Control Group (n₁ = 20)	6.25 ± 1.164	12.95 ± 0.945	11.95 ± 0.887	10.80 ± 0.951	71.99 (<0.001)	430.735 (<0.001)	4.029 (=0.009)
Intervention Group (n₁ = 20)	6.90 ± 0.912	14.75 ± 1.293	14.00 ± 0.725	12.00 ± 1.076			
*t*	1.966	5.028	8.000	3.736			
*p*	0.57	<0.001	<0.001	0.001			

This is the ACP knowledge scores of patients between two groups at different time points.

### Comparison of ACP attitude scores between the two groups

Comparisons of participants’ ACP attitude scores across time points revealed no significant difference between the two groups before intervention (*p* > 0.05). In contrast, at T1, T2, and T3, statistically significant differences in ACP attitude scores were observed between the two groups (*p* < 0.001), as presented in Table 4. The results showed that the ACP attitude scores of patients in both groups changed over time, and there was a significant interaction effect between time and group. Specifically, the ACP attitude scores of the intervention group were significantly higher than those of the control group.

**Table 4:** ACP attitude scores of patients in both groups.

Grouping	Before intervention	After intervention			Between-group effect *F (P)*	Within-group effect *F (P)*	Interaction effect *F (P)*
		Immediate post-intervention	One month post-intervention	Three months post-intervention			
Control Group (n₁ = 20)	40.30 ± 3.181	51.50 ± 5.145	48.35 ± 4.320	45.90 ± 5.261	49.922 (<0.001)	404.648 (<0.001)	55.540 (<0.001)
Intervention Group (n₁ = 20)	40.70 ± 4.305	62.65 ± 4.196	59.50 ± 3.832	56.80 ± 3.172			
*t*	0.334	7.510	8.635	7.935			
*p*	0.74	<0.001	<0.001	<0.001			

This is the ACP attitude scores of patients between two groups at different time points.

### Comparisons of ACP engagement scores between the two groups

Table 5 illustrates the comparative analysis of total scores for ACP participation between the two groups after intervention. Prior to intervention, no statistically significant difference was observed in total engagement scores between the two groups (*p* > 0.05). However, at time points T1, T2, and T3, significant differences were noted in the ACP attitude scores between the two groups (*p* < 0.001). Notably, a significant interaction effect between time and group was identified for ACP engagement scores among participants in both groups (*p* < 0.001). Additionally, the total ACP participation scores in both groups exhibited significant changes over time (*p* < 0.001). Time-related effects differed across groups; specifically, compared with participants in the Control Group (CG), those in the Intervention Group exhibited statistically significant improvements in scores for each dimension of ACP engagement as well as the total score (from baseline to post-intervention).

**Table 5:** Total scores of ACP participation in patients of both groups.

Grouping	Before intervention	After intervention			Between-group effect *F (P)*	Within-group effect *F (P)*	Interaction effect *F (P)*
		Immediate post-intervention	One month post-intervention	Three months post-intervention			
Control Group (n₁ = 20)	36.50 (34.00, 40.05)	63.00 (51.25, 68.00)	56.00 (44.00, 64.75)	53.00 (38.00, 63.50)	25.965 (<0.001)	454.226 (<0.001)	504.720 (<0.001)
Intervention Group (n₁ = 20)	36.00 (35.00, 39.00)	105.00 (76.25, 105.00)	98.00 (67.25, 103.50)	93.00 (66.00, 102.75)			
*z*	0.014	−3.912	−3.750	−3.903			
*p*	0.989	<0.001	<0.001	<0.001			

This is the total scores of ACP participation of patients between two groups at different time points.

## Discussion

### The ACP-T intervention facilitates transitions in participants’ stages of ACP engagement behavior

In the present study, a total of 17 participants exhibited changes in their behavioral stages, with 6 progressing to the contemplation stage and 11 to the preparation stage. These findings demonstrate that the ACP-T intervention effectively promotes transitions in participants’ stages of ACP engagement behavior. Firstly, the ACP-T intervention is grounded in the Trans-theoretical Model of behavior change, which incorporates distinct stage-specific goals and intervention priorities (28). This theoretical foundation enables an in-depth understanding of participants’ diverse stages and needs throughout the process of behavioral change related to ACP engagement. By analyzing factors influencing participants’ behaviors at each stage, the present study developed targeted intervention strategies, thereby facilitating progression from the precontemplation stage to higher stages. For participants with limited ACP knowledge, this study enhanced their awareness and comprehension of advance care planning through knowledge manuals, PPT presentations, and motivational interviewing (29). ACP involves decisions made by participants prior to potential loss of medical decision-making capacity. Given that acute disease onset or re-trauma post-recovery is unforeseeable for participants, this study provided clinical scenarios through methods such as video presentations and case sharing. This approach enabled participants to recognize multiple ACP options for future medical care and enhanced their decision-making capacities (30, 31). Additionally, sharing successful cases was implemented to strengthen their confidence in successful ACP engagement, thereby increasing their participation level and motivation (32). In summary, the present study enhances participants’ involvement in medical decision-making by refining theoretical staging, improving engagement capabilities, and boosting self-efficacy.

### The ACP-T intervention enhances participants’ ACP -related knowledge levels

Following the intervention, statistically significant differences in ACP knowledge scores were observed between groups, within each group, and for the interaction effect—findings confirming the intervention’s effectiveness in improving participants’ ACP knowledge. To promote participants’ comprehensive understanding of ACP, researchers first offered specialized knowledge support. Meanwhile, community healthcare providers leveraged their unique professional strengths—such as established community rapport, local authority, and public credibility—which further facilitated the effective dissemination and participants’ grasp of ACP-related information (33). Grounded in the TTM framework, the present study identified cognitive factors as the primary barriers in the pre-intention stage; accordingly, the goal of ACP intervention in this stage was defined as improving participants’ knowledge levels. Previous research has indicated that ACP-T interventions can enhance cancer participants’ understanding and acceptance of ACP, which plays a critical role in improving end-of-life quality of life (34, 35). In the present study, multiple approaches—including knowledge manuals, group lectures, and video-based education—were employed to enhance participants’ knowledge levels. Specifically, the Handbook of ACP Knowledge was used to reinforce and consolidate ACP knowledge. For group lectures, video and PPT presentations integrating text and images were utilized to deepen participants’ memory retention (32); additionally, community healthcare providers were involved in delivering ACP education to participants (36). By addressing participants’ needs for ACP knowledge and incorporating knowledge review, question-and-answer sessions, and interactive activities at each stage, the ACP-T intervention effectively improved participants’ ACP knowledge levels.

### The ACP-T intervention improves participants’ attitudes toward ACP

The study results showed that after the intervention, statistically significant differences in ACP attitude scores were observed between the two groups (*p* < 0.001), with the intervention group exhibiting significantly higher scores than the control group. These findings indicate that ACP-T participation interventions effectively improve participants’ attitudes toward ACP. In this study, intervention content was linked to traditional Chinese cultural strengths, with communication strategies connecting ACP to Chinese filial piety culture and family values (37). This facilitates emotional resonance, enables participants to correctly understand the significance of ACP, alters their attitudes toward ACP, and promotes the conversion of their ACP behavioral intentions into active willingness. Through discussions with participants about their disease and medical experiences, their perceived needs for ACP were gradually identified. Accumulating evidence from existing studies has demonstrated that intervention programs rooted in the Theory of TTM not only help strengthen participants’ attitudes toward ACP — particularly among individuals diagnosed with cancer — but also contribute to a more adaptive perspective on death (38). In this study, motivational interviewing was integrated into communication strategies, primarily to align with the TTM stages for behavioral changes. A growing body of research has indicated that the application scope of motivational interviewing (MI) has gradually expanded—shifting from traditional inpatient settings to community-family integrated care contexts—and this expansion has been accompanied by demonstrated effectiveness in relevant interventions (39). The intervention enabled older adult participants to perceive ACP benefits, prompting further reflection and thereby promoting their engagement in ACP.

### The ACP-T intervention can enhance participants’ ACP participation

After the intervention, statistically significant differences in total ACP engagement scores were observed between groups, within each group, and for the interaction effect, indicating that the intervention facilitates improved ACP engagement among participants. ACP knowledge levels form the foundation for enhancing engagement. Based on TTM, the intervention program was divided into four distinct stages, with an emphasis on providing tailored information and education at different stages to enhance individuals’ ACP awareness. Personal reflection and decision-making processes are crucial for behavioral change (34). This study utilized diverse intervention formats. Through ACP knowledge explanations, video viewing, case discussions, and exchanges of medical experiences, participants were supported in conducting in-depth reflection on ACP and clarifying their future medical care needs (40). Meanwhile, enhancing self-efficacy is a key factor in promoting ACP engagement (41). In this study, the specific document My Five Wishes was provided to help individuals better understand and navigate the ACP development process. One-on-one motivational interviews were conducted from the 4th to the 6th week of the study, which helped to improve participants’ perceived benefits and strengthen their understanding of ACP (42). In summary, this study can collectively enhance participants’ participation by improving their knowledge, reflection, self-efficacy, and readiness.

### Acceptability and promotability of the ACP-T intervention

The ACP-T intervention in this study has acceptability. Firstly, the TTM theoretical framework in this study provides stage-specific guidance for participants’ ACP engagement. It offers tailored guidance and support based on the distinct stages participants undergo during ACP engagement, facilitating their gradual involvement. Studies have shown that employing scientific methods helps verify effectiveness and feasibility, enhances program credibility, and thereby facilitates participant acceptance (43). This study considers social adaptability and communicates with participants through the concepts of filial piety and family values to generate emotional resonance, thereby enhancing program acceptance. The intervention content primarily involves ACP-related education and is not restricted to specific disease areas. Thus, ACP-T exhibits universal applicability to older adult participants with chronic diseases. This helps reduce family decision-making conflicts and the waste of medical resources, alleviates social pressure and family burdens, and yields significant social benefits (44), thereby enhancing its promotability in community settings.

### Strengths and limitations

In China, current ACP interventions have predominantly centered on participants’ knowledge, attitudes toward death, and related domains, with insufficient attention devoted to ACP engagement. The present study integrates four core dimensions- specifically knowledge, reflection, self-efficacy, and readiness – to incrementally enhance ACP engagement, thereby enriching the evaluative content of ACP clinical practice. The ACP-T program developed in this study harnesses community resources—specifically including healthcare staff from primary care institutions and professional social workers—to deliver comprehensive and continuous medical care services to participants. Concurrently, it is closely aligned with the Theory of TTM; by developing stage-matched intervention strategies tailored to participants’ current readiness levels, the program further facilitates a progressive enhancement in participants’ engagement with ACP. Due to constraints related to human resources, research duration, and other objective factors, the source population of the study was limited, resulting in low sample heterogeneity and insufficient representativeness. However, several limitations of the present study should be explicitly acknowledged. First, the small final sample size (n = 40) may have compromised statistical power, potentially limiting the reliability of the observed results. Second, the single-center study design imposes constraints on the generalizability of the findings to more diverse Chinese community populations. Third, the short follow-up duration (3 months) precludes the ability to draw conclusions regarding the intervention’s long-term efficacy. Despite these limitations, the preliminary findings of this study provide initial evidence supporting the feasibility of implementing such interventions in community health settings. They further underscore the necessity of conducting larger-scale, multi-center trials with extended follow-up periods to validate the intervention’s efficacy more rigorously.

### Implications for research and practice

In the Chinese context, this study developed an ACP-T intervention specifically tailored for community-dwelling older adults with chronic conditions. Beyond providing actionable guidance for primary healthcare institutions, this intervention supports three key objectives: the integration of community resources, the establishment of ACP service pathways, and the exploration of patients’ medical preferences and quality-of-life needs—while simultaneously addressing existing cultural and systemic barriers. The ACP-T intervention is conducive to integrating community resources, assisting community hospitals in establishing ACP service pathways, and exploring the needs of senior community participants regarding future medical preferences, care goals, and quality of life.

Practically, community health workers (CHWs) and primary care teams can integrate ACP promotion into their routine clinical workflows. For instance, during follow-up consultations for patients with chronic conditions, they may distribute ACP educational brochures and conduct preliminary ACP assessments. Furthermore, the use of culturally adapted resources—such as family-oriented discussion guides—can help alleviate taboos surrounding end-of-life discussions. Equally importantly, collaboration with local community-based organizations to deliver phased ACP education programs is essential to enhance reach and acceptance. This comprehensive strategy not only addresses key cultural and systemic barriers but also strengthens the long-term sustainability of ACP initiatives within community healthcare contexts.

## Conclusion

The ACP-T program developed in this study can leverage community resources to provide care-related support for participants, with the potential to facilitate continuous service delivery. In this feasibility trial, the preliminary implementation of the intervention program demonstrated promising effects in improving the target population’s knowledge of, attitudes toward, and engagement in ACP. These findings provide initial insights into the program’s potential applicability within community-based settings. Notably, by enhancing participants’ ACP engagement, the program may facilitate respect for their medical autonomy; however, claims regarding its broader impacts—such as the alleviation of social pressure or family care burdens—were not directly assessed in this study and thus require cautious interpretation. While these preliminary observations suggest potential benefits of the program, they necessitate further validation in larger-scale, multi-center trials. Additionally, the program’s value should be contextualized within the inherent limitations of this feasibility study.

## Data Availability

The original contributions presented in the study are included in the article/supplementary material, further inquiries can be directed to the corresponding author.

## References

[1] Tu WJ Zeng X Liu Q. Aging tsunami coming: the main finding from China's seventh national population census. Aging Clin Exp Res. (2022) 34:1159–63. doi: 10.1007/s40520-021-02017-4, PMID: 34727357

[2] Yee CJ Penumudi A Lewinson T Khayal IS. End-of-life Cancer care interventions for racially and ethnically diverse populations in the USA: a scoping review. Cancers (Basel). (2025) 17:2209. doi: 10.3390/cancers17132209, PMID: 40647507 PMC12249464

[3] Gravante F Trotta F Latina S Simeone S Alvaro R Vellone E . Quality of life in ICU survivors and their relatives with post-intensive care syndrome: a systematic review. Nurs Crit Care. (2024) 29:807–23. doi: 10.1111/nicc.13077, PMID: 38622971

[4] Dinescu A. Advance care planning. Clin Geriatr Med. (2021) 37:605–10. doi: 10.1016/j.cger.2021.06.001, PMID: 34600725

[5] Sudore RL Lum HD You JJ Hanson LC Meier DE Pantilat SZ . Defining advance care planning for adults: a consensus definition from a multidisciplinary Delphi panel. J Pain Symptom Manag. (2017) 53:821–832.e1. doi: 10.1016/j.jpainsymman.2016.12.331, PMID: 28062339 PMC5728651

[6] Metzger E. Advances in advance directives. Am J Geriatr Psychiatry. (2022) 30:624–6. doi: 10.1016/j.jagp.2022.02.006, PMID: 35277323

[7] Sedini C Biotto M Crespi Bel’skij LM Moroni Grandini RE Cesari M. Advance care planning and advance directives: an overview of the main critical issues. Aging Clin Exp Res. (2022) 34:325–30. doi: 10.1007/s40520-021-02001-y 34655048 PMC8847241

[8] Lim MK Wong PS Othman S Mohd Mydin FH Lim PS Lai PSM. A systematic review of non-seriously ill community-dwelling Asians' views on advance care planning. J Am Med Dir Assoc. (2023) 24:1831–42. doi: 10.1016/j.jamda.2023.09.008, PMID: 37844872

[9] Meghani SH Hinds PS. Policy brief: the Institute of Medicine report dying in America: improving quality and honoring individual preferences near the end of life. Nurs Outlook. (2015) 63:51–9. doi: 10.1016/j.outlook.2014.11.007, PMID: 25645482

[10] National Academies of Sciences EAM, Medicine HA, Division, Policy BOHS, Services BOHC, Illness ROQC. The challenges and opportunities of advance care planning: Proceedings of a workshop. Washington (DC): National Academies Press (US) (2021). 33886174

[11] Korfage IJ Carreras G Arnfeldt CC Billekens P Bramley L Briggs L . Advance care planning in patients with advanced cancer: a 6-country, cluster-randomised clinical trial. PLoS Med. (2020) 17:e1003422. doi: 10.1371/journal.pmed.1003422 33186365 PMC7665676

[12] Ng R Lip HK Lim J Weifen L. Advance care planning in Singapore: the genesis and evolution of a national programme. Z Evid Fortbild Qual Gesundhwes. (2023) 180:99–102. doi: 10.1016/j.zefq.2023.05.018 37407336

[13] Ding J Cook A Saunders C Chua D Licqurish S Mitchell G . Uptake of advance care planning and its circumstances: an nationwide survey in Australian general practice. Health Soc Care Community. (2022) 30:1913–23. doi: 10.1111/hsc.13570, PMID: 34529292

[14] Zhu T Zhang J Shi Y Yi J Zhang Q Zhao Y . Awareness and attitudes toward advance care planning among community-dwelling older adults in China: a mixed-methods study. Am J Hosp Palliat Care. (2020) 37:743–9. doi: 10.1177/1049909120905255, PMID: 32052643

[15] Fried TR Redding CA Robbins ML Paiva AL O'Leary JR Iannone L. Development of personalized health messages to promote engagement in advance care planning. J Am Geriatr Soc. (2016) 64:359–64. doi: 10.1111/jgs.13934, PMID: 26804791 PMC4760894

[16] Kotwal AA Barnes DE Volow AM Li BH Boscardin WJ Sudore RL. Engaging diverse older adults with cognitive impairment and caregivers in advance care planning: a pilot study of the interactive PREPARE website. Alzheimer Dis Assoc Disord. (2021) 35:342–9. doi: 10.1097/WAD.0000000000000465, PMID: 34310443 PMC8604734

[17] Nouri S Street RJ Jr Barnes DE Shi Y Volow AM Li B . Empowering patients with the PREPARE advance care planning program results in reciprocal clinician communication. J Am Geriatr Soc. (2022) 70:585–91. doi: 10.1111/jgs.17540 34758115 PMC8821241

[18] Song D Yu T Zhi S Chang C Sun J Gao S . Experiences and perspectives on the optimal timing for initiating advance care planning in patients with mild to moderate dementia: a meta-synthesis. Int J Nurs Stud. (2024) 154:104762. doi: 10.1016/j.ijnurstu.2024.104762, PMID: 38613968

[19] Liu X Chen H Zhang L Zhang Q Feng T Liu D. Advance care planning engagement among family members of community-dwelling elderly patients with chronic diseases in China: a mixed-methods study. J Hosp Palliat Nurs. (2022) 24:E26–34. doi: 10.1097/NJH.0000000000000829, PMID: 35045050

[20] Tanaka GM Efstathiou N Innes R Metaxa V. End-of-life care in the intensive care unit. Anaesthesia. (2023) 78:636–43. doi: 10.1111/anae.15908 36633479

[21] Song D. Interventions to promote readiness for advance care planning: a systematic review and meta-analysis. Int J Nurs Stud. (2025) 161:104957 39541639 10.1016/j.ijnurstu.2024.104957

[22] Hoffmann F Schnakenberg R Silies K Berg A Kirchner Ä Jaschke J . Effects of advance care planning in care dependent community-dwelling older persons (STADPLAN): a cluster-randomised controlled trial. Palliat Med. (2023) 37:1193–201. doi: 10.1177/02692163231180322, PMID: 37310014 PMC10503242

[23] Pilch M Lunt V May P Mockler D Thomas S Doyle F. Facilitators and barriers to stakeholder engagement in advance care planning for older adults in community settings: a hybrid systematic review protocol. HRB Open Res. (2020) 3:38. doi: 10.12688/hrbopenres.13082.2, PMID: 34212126 PMC8212429

[24] Pairojkul S Raksasataya A Sorasit C Horatanaruang D Jarusomboon W. Thailand's experience in advance care planning. Z Evid Fortbild Qual Gesundhwes. (2023) 180:85–9. doi: 10.1016/j.zefq.2023.05.010, PMID: 37400279

[25] Tan G Quek G Lum N Tan GWH Quek GSM Lum NJX . Validation of the advance care planning engagement survey in Singapore. BMC Palliat Care. (2025) 24:11. doi: 10.1186/s12904-024-01640-y, PMID: 39794785 PMC11720953

[26] Lai PS Mohd MS Chinna K Othman S. The development and validation of the advance care planning questionnaire in Malaysia. BMC Med Ethics. (2016) 17:61. doi: 10.1186/s12910-016-0147-8 27756366 PMC5069889

[27] Sudore RL Stewart AL Knight SJ McMahan RD Feuz M Miao Y . Development and validation of a questionnaire to detect behavior change in multiple advance care planning behaviors. PLoS One. (2013) 8:e72465. doi: 10.1371/journal.pone.0072465, PMID: 24039772 PMC3764010

[28] Liu Y Zhou Y Li X Zhang J. Influences of trans-theoretical model-based diet nursing intervention on sarcopenia and quality of life in maintenance hemodialysis patients. Nutr Hosp. (2024) 41:278–85. doi: 10.20960/nh.04844, PMID: 38328951

[29] Matthieu MM Oliver CM Hernandez GI McCullough JA Adkins DA Mallory MJ . Application of motivational interviewing to group: teaching advance care planning via group visits for clinical professionals. Patient Educ Couns. (2024) 120:108116. doi: 10.1016/j.pec.2023.108116, PMID: 38150951

[30] Shu X Chen Q Zhou Y Yang Z Zhang Q. The effectiveness of video decision aid on advance care planning with adult patients: a systematic review and Meta-analysis of randomized controlled trials. J Hosp Palliat Nurs. (2023) 25:E8–E13. doi: 10.1097/NJH.0000000000000919, PMID: 36348512

[31] Volandes AE Zupanc SN Paasche-Orlow MK Lakin JR Chang Y Burns EA . Association of an Advance Care Planning Video and Communication Intervention with Documentation of advance care planning among older adults: a nonrandomized controlled trial. JAMA Netw Open. (2022) 5:e220354. doi: 10.1001/jamanetworkopen.2022.0354, PMID: 35201306 PMC8874350

[32] Lamoureux ET Jacelon C. Motivational interviewing, readiness for change, walking, and functional ability in older adults. J Gerontol Nurs. (2022) 48:23–9. doi: 10.3928/00989134-20220209-04, PMID: 35201925

[33] Korkmaz Yaylagul YN Demirdas FB Melo P Silva R. Opinions of older individuals on advance care planning and factors affecting their views: a systematic review. Int J Environ Res Public Health. (2023) 20:5780. doi: 10.3390/ijerph20105780 37239509 PMC10218071

[34] Levoy K Salani DA Buck H. A systematic review and gap analysis of advance care planning intervention components and outcomes among Cancer patients using the Transtheoretical model of health behavior change. J Pain Symptom Manag. (2019) 57:118–39. doi: 10.1016/j.jpainsymman.2018.10.502, PMID: 30595148

[35] Zhu T Martina D Heide A V Heide Agnes van der Korfage Ida J Rietjens Judith AC. The role of acculturation in the process of advance care planning among Chinese immigrants: a narrative systematic review Palliat Med 2023 37 1063–1078. doi: 10.1177/02692163231179255, PMID: 37309994 PMC10503260

[36] Jeong S Cleasby P Ohr SO Barrett T Davey R Oldmeadow C. Efficacy of normalisation of advance care planning (NACP) for people with chronic diseases in hospital and community settings: a quasi-experimental study. BMC Health Serv Res. (2021) 21:901. doi: 10.1186/s12913-021-06928-w, PMID: 34470636 PMC8408987

[37] Michael N O'Callaghan C Baird A Gough K Krishnasamy M Hiscock N . A mixed method feasibility study of a patient- and family-centred advance care planning intervention for cancer patients. BMC Palliat Care. (2015) 14:27. doi: 10.1186/s12904-015-0023-1 25981642 PMC4456060

[38] Canny A Mason B Stephen J Hopkins S Wall L Christie A . Advance care planning in primary care for patients with gastrointestinal cancer: feasibility randomised trial. Br J Gen Pract. (2022) 72:e571–80. doi: 10.3399/BJGP.2021.0700, PMID: 35760566 PMC9242676

[39] Galbraith N Rose C Rose P. The roles of motivational interviewing and self-efficacy on outcomes and cost-effectiveness of a community-based exercise intervention for inactive middle-older aged adults. Health Soc Care Community. (2022) 30:e1048–60. doi: 10.1111/hsc.13510, PMID: 34260782

[40] Ufere NN Robinson B Donlan J Indriolo T Bloom J Scherrer A . Pilot randomized controlled trial of an advance care planning video decision tool for patients with advanced liver disease. Clin Gastroenterol Hepatol. (2022) 20:e3:2287–95. doi: 10.1016/j.cgh.2021.10.027, PMID: 34718173

[41] Murray AN Monahan K Sacco A Paiva A Redding C Robbins M. Development and validation of a measure of self-efficacy for advance care planning. Am J Hosp Palliat Care. (2024) 41:873–81. doi: 10.1177/10499091231210504, PMID: 37879089

[42] Fried TR Yang M Martino S Iannone L Zenoni M Blakley L . Effect of computer-tailored print feedback, motivational interviewing, and motivational enhancement therapy on engagement in advance care planning: a randomized clinical trial. JAMA Intern Med. (2022) 182:1298–305. doi: 10.1001/jamainternmed.2022.5074, PMID: 36342678 PMC9641591

[43] Mochizuki T Yamashita D Miura C Nakamura M Izumi S(S). Feasibility and acceptability of advance care planning facilitated by nonphysician clinicians in Japanese primary care: implementation pilot study. J Gen Fam Med. (2023) 24:30–7. doi: 10.1002/jgf2.586, PMID: 36605916 PMC9808147

[44] Rizzo VM Engelhardt J Tobin D Penna RD Feigenbaum P Sisselman A . Use of the stages of change transtheoretical model in end-of-life planning conversations. J Palliat Med. (2010) 13:267–71. doi: 10.1089/jpm.2009.0281, PMID: 20078215

